# Replicating finding, answering questions: closer to the truth about COVID-19 associated VAP

**DOI:** 10.1186/s13054-023-04476-9

**Published:** 2023-06-05

**Authors:** Charles-Hervé Vacheron, Romain Fort, Tristan Dagonneau, Arnaud Friggeri, Florent Wallet, HospiceRea Study Group, Julien Bohe, Jean-christophe Richard, Thomas Rimmele, Anne-Claire Lukaszewicz, Frederic Dailler, Frederic Aubrun, Jean-Luc Fellahi, Bernard Allaouchiche, Elisabeth Hodille, Melanie Levrard, Chloé Gerbaud-Coula, Emilie Joffredo, Fabrice Thiolliere, Olivia Vassal, Donatien de Marignan, Lucille Jay, Vincent Piriou, Arnaud Friggeri, Florent Wallet

**Affiliations:** 1 PHE3ID, Centre International de Recherche en Infectiologie, Institut National de la Santé et de la Recherche Médicale U1111, CNRS Unité Mixte de Recherche 5308, École Nationale Supérieure de Lyon, Université Claude Bernard Lyon 1, Villeurbanne, France; 2 Département d’Anesthésie Réanimation-Medecine Intensive, Centre Hospitalier Lyon Sud, Hospices Civils de Lyon, 165 Chemin du Grand Revoyet, 69310 Pierre-Bénite, France; 3 Université Lyon 1, Lyon, France; 4 Département d’information Médical, Hospices Civiles de Lyon, Lyon, France; 5 RESHAPE Research on Healthcare Performance, U1290, Université Claude Bernard Lyon 1, Lyon, France

## Correspondance

In their recent comment, Shorr and Zilberberg registered concerns about the analysis performed concerning the association of Ventilator-associated pneumonia (VAP) and steroids in patients with COVID-19 [1]. They discussed the discrepancy between the results of three studies that found an association or none between VAP and steroids [2–4]. The three clinical studies used three different methods and a set of variables for adjustment. In the Scaravilli et al*.* work, 158 patients with steroids were matched 1:1 to patients without steroids using propensity score matching on 9 variables, and then, a univariate competing risk analysis was performed (*propensity matching*). They found a higher risk of VAP incidence in the steroid group (subdistribution Hazard Ratio (sHR) 1.81[1.31;2.50]). Lamouche-Wilquin et al. used a retrospective cohort of patients comparing patients with early corticosteroid therapy (< 5 days—369 patients) with no or late steroid therapy (301 patients) [4]. In their work, they performed a competing risk analysis (Fine and Gray model) with adjustment for unbalanced variables in the univariate analysis, finally selecting 3 variables: age, body mass index, and Charlson's comorbidity index (*Competing Risk Regression*). They found a smaller but still statistically significant-effect of steroids on the occurrence of VAP (sHR 1.28[1.03–1.58]. Finally, Saura et al. used a retrospective cohort of 354 patients without steroid therapy, comparing them to 191 patients who used steroid therapy during their ICU stay, not limited to the use of dexamethasone to treat COVID. They used multivariate cause-specific Cox's proportional hazard models with adjustment for pre-specified confounders: age, sex, BMI, SAPS II, MacCabe classification, immunosuppression, recent hospitalization, recent antibiotics, shock, ARDS and cardiac arrest (*cause-specific Cox model*). They found no association between steroids and VAP; however, there appeared to be a temporal interaction between steroid use and VAP (cause-specific Hazard Ratio (csHR) 0.47[0.17;1.31] at day 2, and 1.94[1.09;3.46] at day 21, *p value* for the overall effect 0.08). Two studies used prior knowledge to select variables, and one was based on univariate analysis. Although all methods are commonly used in the literature, univariate analysis is regularly pointed out as a highly biased method to select variables [5]. Shorr et al. emphasized the importance of the model used to model the influence of co-variable on the occurrence of VAP and the importance of the competing risk. We hypothesize that the model is less important than the variable used to perform the adjustment – or matching of the groups.

Therefore, we used a cohort from an unpublished work, a French multicenter retrospective cohort, including patients admitted to the ICU for SARSq-CoV-2 and receiving mechanical ventilation for more than 48 h. Two groups were constituted: patients mainly before June 2020 who did not benefit from dexamethasone and patients who received steroids according to the RECOVERY study. For these patients, several characteristics were extracted from the electronic health record, by manual extraction with return to the patient file. VAP was defined by the presence of microbiologic documentation and clinical signs (radiological criteria were not retained due to the high variability of the imaging criteria in COVID-19 patients). We then performed the 3 different methods described above 2 times: once by using the variable available in our cohort or a surrogate (6/9, 3/3, and 8/11, respectively, for the studies of Scaravilli, Lamouche-Wilquin, and Saura) (the Minimal model), and by using the maximum available adjustment criteria used among the 3 studies, resulting in 10 criteria: Age, Sex, BMI, SAPS II, Charlson Comorbidity Index, Immunosuppression, Shock, ARDS, PaO_2_/FiO_2_, and SOFA Score (Full Model) (details of the statistical plan available in Additional file 1: Materials 1 and 2). There was no major difference in modeling, except for steroid therapy as a time-dependent variable, as our patients received early steroid therapy in our cohort, and it was therefore not a time-dependent variable.

Finally, 1080 patients were included, 70% of whom had dexamethasone therapy. Our group was unbalanced on BMI, immunosuppression, and severity on admission (SOFA, SAPS II, and PaO2/FiO2). Patients treated with dexamethasone presented more ARDS (Additional file 1: Material 3), had more VAP compared to the control group (59 vs 51%, p = 0.023), and were more often mechanically ventilated. Regarding the minimal model, both the use of propensity matching and competing risk regression found an association between steroid use and VAP (sHR 1.27 [1.07;1.51] and 1.24 [1.03;1.48], respectively). Using the full model, no association was found between steroid use and VAP (with HR ranging from 1.04 [0.87;1.25] to 1.12 [0.93;1.36]) (Table 1; detailed results in Additional file 1: Material 2).

**Table 1:** Impact of steroid depending on the model and the variable used

	Derived from the study	Minimal model	Full model
Propensity matching	1.81 [1.31;2.50]	1.27 [1.07;1.51]	1.09 [0.87;1.36]
Competing risk regression	1.28 [1.03–1.58]	1.24 [1.03;1.48]	1.04 [0.87;1.25]
cs Cox model	–*	1.13 [0.93;1.36]	1.12 [0.93;1.36]

Cs: Cause specific, Minimal model: including the variable available used in the original study, Full model: using all variable available

*No csHR estimated for the overall effect of steroid

Despite our different viewpoints to explain the heterogeneity of the results across studies, we agree with Shorr and Zilberberg's conclusion: even if the use of corticosteroids is associated with a higher incidence of VAP, the strength of the association is probably very small, and the clinician should not limit its appropriate use.

## Supplementary Information

**Additional file 1**. **Supplementary Material 1**: Description of the method used by the 3 studies. **Supplementary Material 2**: Statistical plan and detailed results. **Supplementary Material 3**: Description of the patients.

## Data Availability

The datasets generated and/or analyzed during the current study are not publicly available due French legislation.
