# Supplementary material for: Replicating finding, answering questions: closer to the truth about COVID-19 associated VAP
Source: Crit Care. 2023 Jun 5;27:220. doi: 10.1186/s13054-023-04476-9 (PMC10240453; doi:10.1186/s13054-023-04476-9)
Supplement: Supplementary file 1 — Additional file 1. Supplementary Material 1: Description of the method used by the 3 studies. Supplementary Material 2: Statistical plan and detailed results. Supplementary Material 3: Description of the patients. [file 13054_2023_4476_MOESM1_ESM.docx]

**Supplementary Material 1: Description of the method used by the 3 studies.**

|  | **Method** | **Variable for adjustment** | **Variable available** |
| --- | --- | --- | --- |
| Scaravilli | Propensity score matching  Univariate competing risk regression | 9 variables   - Age - Sex - Weight - PEEP Level - PaO2/FiO2 - non-respiratory SOFA score - Charlson Comorbidity Index - C-reactive protein concentration - Hospital admission | 6 variables   - Age - Sex - BMI - - - PaO2/FiO2 - SOFA Score - Charlson Comorbidity Index (CCI) - - - - |
| Lamouche-Wilquin | Adjusted Competing risk regression | 3 variables   - Age - BMI - Charlson Comorbidity Index | 3 variables   - Age - BMI - Charlson Comorbidity Index |
| Saura | Cause specific Cox proportional hazard models | 11 variables   - Age - Sex - BMI - SAPS II - MacCabe classification - Immunosuppression - Recent hospitalization - Recent antibiotics - Shock - ARDS - Cardiac arrest. | 8 variables   - Age - Sex - BMI - SAPS II - Charlson Comorbidity Index - Immunosuppression* - - - - - Shock* - ARDS* - - |

**Supplementary Material 2 : Statistical plan and detailed results**

Variables were expressed as median [interquartile range, IQR], or count (percentage). Univariate analyses were performed using the Wilcoxon, Chi^2^, or Fisher test according to the type of variable (quantitative or qualitative, respectively). Missing variables were imputed using *Multivariate Imputation by Chained Equations*, using 20 chained equations, as described in the method of Saura et al. For every model below, the censoring date of the patient was at 30^th^ day, and the competing events were extubation or death without VAP.

**Cause-specific Cox regression model**

The association between corticosteroids and VAP was modeled using cause specific Cox regression . Hazard assumption proportionality of corticosteroids and of the full models was assessed by using the scaled Schoenfeld residuals plots and was satisfied in every model performed.

|  | csHR | *p value* |
| --- | --- | --- |
| **Univariate model** |  |  |
| Dexamethasone | 1.14 [0.95;1.36] | 0.151 |
| **Model from Saura et al** |  |  |
| Dexamethasone | 1.13 [0.93;1.36] | 0.211 |
| Age | 0.99 [0.98;1.00] | 0.093 |
| BMI | 1.02 [1.00;1.03] | 0.024 |
| SAPS II | 1.00 [1.00;1.00] | 0.956 |
| Sex (Male) | 1.35 [1.12;1.63] | 0.002 |
| Shock | 1.52 [0.99;2.35] | 0.057 |
| ARDS | 1.15 [0.88;1.50] | 0.312 |
| Charlson Comorbidity Index | 0.99 [0.94;1.04] | 0.675 |
| Immunosuppression | 0.81 [0.59;1.11] | 0.193 |
| **Full Model** |  |  |
| Dexamethasone | 1.12 [0.93;1.36] | 0.229 |
| Age | 0.99 [0.98;1.00] | 0.109 |
| BMI | 1.02 [1.00;1.03] | 0.037 |
| SAPS II | 1.00 [0.99;1.00] | 0.722 |
| Sex (Male) | 1.36 [1.12;1.64] | 0.002 |
| Shock | 1.51 [0.98;2.33] | 0.064 |
| ARDS | 1.14 [0.86;1.50] | 0.374 |
| Charlson Comorbidity Index | 0.99 [0.94;1.04] | 0.064 |
| Immunosuppression | 0.81 [0.60;1.11] | 0.199 |
| PaO_2_/FiO_2_ | 1.00 [1.00;1.00] | 0.627 |
| SOFA | 1.01 [0.99;1.03] | 0.533 |

csHR : Cause specific Hazard Ratio

**Propensity score**

A propensity scores matching procedure, with a 1:1 ratio was applied to identify two cohorts of patients matched based on the covariates mentioned in Supplementary Material 2. The balancement of both cohorts created (one created using the covariate of Scaravilli et l, one with the full model) was assessed using standardized mean deviation (SMD).

Using the variable from Scaravilli et al, 300 patients were matched in each group (caliper value of 0.1), and all SMD were below 0.1 except the BMI (0.11). Using the 10 variables, and a caliper value of 0.1, 281 patients were matched. All SMD were below 0.1 except the SAPS II (0.14). Given this low difference of theses variable, and the minimal impact of these covariable on the occurrence of VAP, the balancement of cohort was judged satisfying. Then, competing risk analysis was performed using the Fine and Gray model to estimate sub-hazard ratio

|  | sHR | *p value* |
| --- | --- | --- |
| **Model using Scaravilli variable for the matching** | |  |
| Dexamethasone | 1.27 [1.07;1.51] | 0.007 |
| **Model using all variable for the matching** | |  |
| Dexamethasone | 1.09 [0.87;1.36] | 0.450 |

sHR : subdistribution Hazard Ratio

**Standard Competing risk analysis**

A standard competing risk analysis was performed, using the Fine and Gray model, using solely the dexamethasone status, the variable included int the model from Lamouche-Wilquin et al, and the full set of variables.

|  | sHR | *p value* |
| --- | --- | --- |
| **Univariate model** |  |  |
| Dexamethasone | 1.27 [1.07;1.51] | 0.007 |
| **Model from Lamouche-Wilquin** | |  |
| Dexamethasone | 1.24 [1.03;1.48] | 0.017 |
| Age | 0.99 [0.98;1.00] | 0.100 |
| BMI | 1.02 [1.00;1.03] | 0.002 |
| **Full Model** |  |  |
| Dexamethasone | 1.04 [0.87;1.25] | 0.651 |
| Age | 0.99 [0.95;1.00] | 0.089 |
| BMI | 1.02 [1.00;1.03] | 0.104 |
| SAPS II | 0.99 [0.99;1.00] | 0.509 |
| Sex (Male) | 1.40 [1.17;1.69] | <0.001 |
| Shock | 2.78 [1.79;4.33] | <0.001 |
| ARDS | 2.33 [1.73;3.14] | <0.001 |
| Charlson Comorbidity Index | 0.86 [0.63;1.16] | 0.357 |
| Immunosuppression | 0.86 [0.63;1.16] | 0.329 |
| PaO_2_/FiO_2_ | 0.99 [0.99;1.00] | 0.436 |
| SOFA | 1.00 [0.98;1.02] | 0.885 |

sHR : subdistribution Hazard Ratio

Analyses were conducted using R software version 3.6.4, and using the package mice, survival, MatchIt, and cmprsk.

**Supplementary Material 3 : Description of the patients**

|  | No Dexamethasone  n=329 | Dexamethasone  n=751 |  |
| --- | --- | --- | --- |
| Age, years | 67 [58-74] | 68 [59-74] | 0.125 |
| BMI, kg/m^2^ | 28 [24-32] | 29 [26-33] | <0.001 |
| Sex, male | 244 (74%) | 520 (69%) | 0.118 |
| Charlson Comorbidity Index | 4 [2-5] | 4 [2-5] | 0.701 |
| Immunosuppression | 22 (7%) | 93 (12%) | 0.007 |
| **Severity at admission** |  |  |  |
| SOFA | 8 [4-12] | 7 [3-10] | <0.001 |
| SAPS II | 45 [32-63] | 39 [29-52] | <0.001 |
| PaO2/FiO2 | 87 [68-130] | 73 [62-97] | <0.001 |
| **Characteristic of the ICU stay** | |  |  |
| Shock | 293 (89%) | 688 (91%) | 0.221 |
| ARDS | 201 (61%) | 630 (84%) | <0.001 |
| **Outcome** |  |  |  |
| Number of patients with at least one VAP | 168 (51%) | 441 (59%) | 0.023 |
| Duration of mechanical ventilation | 13 [4-25] | 15 [8-28] | 0.003 |
| Length of ICU stay | 18 [7-30] | 19 [11-33] | 0.004 |
| Mortality | 142 (43%) | 309 (41%) | 0.581 |
